# Smoking reduction using electronic nicotine delivery systems in combination with nicotine skin patches

**DOI:** 10.1007/s00213-023-06401-y

**Published:** 2023-07-17

**Authors:** Jed E. Rose, Suzanne Frisbee, David Campbell, Alfred Salley, Susan Claerhout, James M. Davis

**Affiliations:** 1 Duke Center for Smoking Cessation, Duke University School of Medicine, 2424 Erwin Road, Suite 201, Durham, NC 27705 USA; 2 Duke Cancer Institute, Durham, NC USA

**Keywords:** Cigarette, Electronic nicotine delivery system, ENDS, E-cigarette, Smoking, Smoking cessation, Harm reduction, Nicotine patch

## Abstract

**Abstract:**

**Rationale:**

Electronic nicotine delivery systems (ENDS) are used by smokers seeking to reduce combustible cigarette (CC) use, but the role of nicotine replacement vs. behavioral and sensory factors is still poorly understood. We hypothesized that providing nicotine from ENDS in addition to nicotine skin patches would promote smoking reduction relative to non-nicotine control ENDS.

**Objectives:**

To assess the effects on smoking behavior of using nicotine vs. placebo ENDS in smokers using nicotine vs. placebo patches.

**Methods:**

Ninety-four daily smokers were enrolled in a study that randomly assigned them to receive ENDS with nicotine vs. without nicotine and skin patches with vs. without nicotine. Smoking reduction and cessation were assessed over an 8-week period by self-report and by expired air carbon monoxide (CO) measurements. The primary outcome was defined as reduction in expired air CO.

**Results:**

The use of nicotine in ENDS led to significant reductions in smoking (ENDS nicotine vs. placebo difference in CO change = −9.2 ppm; 90% CI (−1.5 ppm, −16.9 ppm)) and was highly correlated with reductions in self-reported cigarettes per day (*r*=0.6). The effect of nicotine in nicotine patches was not statistically significant (patch nicotine vs. placebo difference in CO change = −0.1 ppm; 90% CI (−7.8 ppm, 7.6 ppm)).

**Conclusions:**

The presence of nicotine in ENDS was associated with a large reduction in smoking. Additional studies will be needed to determine whether there may be additive effects of nicotine ENDS and nicotine patches on smoking abstinence.

## Introduction

The burden of disease and death attributable to combustible cigarette (CC) smoking is enormous, with an estimated 540,000 premature deaths annually in the USA and over 7 million deaths worldwide (Carter et al. 2015; GBD 2019 Risk Factors Collaborators 2020). Considerable progress has been made toward reducing the prevalence of smoking, through education about the harms of smoking, increased taxation/regulation, and the greater availability of cessation treatments (Centers for Disease Control 2014; Chaloupka et al. 2012). However, despite a gradual reduction in smoking prevalence over the years, in 2020, 12.5% of the adult US population, or 35 million individuals, continued to smoke (Cornelius et al. 2022).

Tobacco harm reduction (THR) refers to reducing a tobacco user’s exposure to tobacco toxins short of complete abstinence and has emerged as a commonly used complementary strategy to cessation efforts (Warner 2019). Although the most desirable goal is always complete tobacco cessation, this is not always feasible, and reduced exposure to tobacco toxins is associated with demonstrable health benefits (Rodu and Godshall 2006). Electronic nicotine delivery systems (ENDS), e.g., e-cigarettes, are accepted by a large portion of the European medical community as a legitimate treatment for smoking (Medicines and Healthcare Products Regulatory Agency 2021). In the USA, ENDS are not widely accepted by the medical community as a treatment for smoking (Hurst et al. 2018), but ENDS are nonetheless used by many smokers in the USA to reduce or quit smoking (Kasza et al. 2022).

Although ENDS produce a non-zero level of toxins and long-term health effects are still being evaluated, there appears to be growing agreement that ENDS produce significantly lower levels of toxins than cigarettes (Goniewicz et al. 2014). According to the 2010 US Surgeon General’s Report (Centers for Disease Control and Prevention 2010) and other expert reviews, tobacco combustion is implicated in generating the toxins that are the major cause of smoking-related diseases. Instead of relying on combustion of tobacco which occurs at high temperatures (e.g., 800 C), ENDS heat a solution (usually a mixture of propylene glycol, glycerol, nicotine, and flavorings) at a lower temperature (e.g., 200 C) resulting in a nicotine-containing aerosol (Geiss et al. 2016). Compared to cigarette smoke, measures of constituents in ENDS aerosol show substantial reductions in most toxicants, including carbon monoxide, carcinogens (including tobacco-specific nitrosamines and polycyclic aromatic hydrocarbons), and volatile organic compounds such as acrolein, acrylamide, acrylonitrile, 1,3-butadiene, and ethylene oxide (McRobbie et al. 2015; Tayyarah and Long 2014). Thus, the Royal College of Physicians (Royal College of Physicians (London) and Tobacco Advisory Group 2016) and the National Academies of Sciences, Engineering, and Medicine (2018) concluded that transition from inhalation of smoke from tobacco combustion to aerosol from ENDS results in a reduction in health risks to smokers. In accordance with this harm reduction perspective, the FDA recently authorized the marketing of an e-cigarette, with the conclusion that it was “appropriate for the protection of the public health” (U.S. Food and Drug Administration 2021).

Regarding ENDS effects on smoking reduction or cessation, ENDS are hypothesized to be effective substitutes for combustible cigarettes because they provide nicotine replacement and also because they provide a substitute for behavioral, sensory, or ritual cues driving smoking (Di Piazza et al. 2020; Johnson et al. 2022). Previous studies have shown that smokers who are offered ENDS as an alternative to cigarettes show substantial reductions in cigarette use. A Cochrane Collaboration Review (McRobbie et al. 2014) summarized evidence that nicotine-containing ENDS were significantly more effective than placebo in producing at least 50% reductions in smoking (36% of smokers reduced smoking by at least half compared to 27% with placebo: *RR* 1.31, 95% CI 1.02 to 1.68). Moreover, nicotine-containing ENDS were more effective than nicotine skin patches in reducing cigarette use (61% of smokers reduced smoking by at least half using ENDS vs. 44% with nicotine patch (*RR* 1.41, 95% CI 1.20 to 1.67). A recent Cochrane Collaboration Review (Hartmann-Boyce et al. 2022) concluded that ENDS increase quit rates more than nicotine replacement therapy (NRT) (*RR* 1.63, 95% CI: 1.30–2.04, based on six studies with 2378 participants). In the largest comparative trial thus far, ENDS were found to be more efficacious than NRT (largely combination NRT), with biochemically confirmed smoking abstinence rates of 18% and 9.9% at 1 year, respectively (Hajek et al. 2019). Although these findings show promise for ENDS as a treatment for cigarette smoking, recent observational studies of smokers using ENDS without medical guidance indicate that over 50% of these unassisted quit attempts resulted in the combined use of cigarettes and ENDS (Martinez et al. 2021), a practice leading to higher toxin exposure than the use of ENDS alone (Goniewicz et al. 2018).

In order to understand treatment effects of ENDS, it is necessary to understand the contribution and potential shortcomings of nicotine replacement provided by ENDS. Cigarette smoking results in typical peak arterial plasma nicotine concentrations of 40–50 ng/mL (Guthrie et al. 2004; Henningfield 1993). In between peaks, many smokers maintain “trough” venous plasma nicotine levels of 15–20 ng/mL (McNabb 1982). A nicotine patch replicates the typical trough plasma nicotine levels of smoking but does not provide the peak nicotine levels that many smokers appear to require to experience nicotine reward (Rasmussen et al. 2018). Most current ENDS products provide peak nicotine concentrations that are significantly lower than those obtained from smoking conventional cigarettes (Goldenson et al. 2021a, b). Thus, the combination of nicotine patch plus ENDS might yield nicotine pharmacokinetics that is more similar to cigarette smoking, by raising both the peak arterial and trough venous nicotine concentrations normally observed with the use of ENDS. Thus, it seems plausible that the combination of steady-state nicotine delivery from a patch and on-demand rapid nicotine delivery from ENDS could allow a smoker to quit more easily than when using nicotine patch or ENDS alone. This treatment principle is seen in the well-established practice of combining nicotine patch treatment with short-acting nicotine formulations such as nicotine gum or lozenge, or rapid-acting nasal spray, and has been shown to increase abstinence rates compared to patch alone (Blondal et al. 1999; Wadgave and Nagesh 2016).

There is currently little information about the relative contribution of ENDS nicotine content in smoking reduction or cessation and the use of ENDS in the context of using other nicotine containing products such as nicotine skin patches. In this study, we explored the potential usefulness of combining ENDS with nicotine patches and hypothesized that the use of nicotine vs. no nicotine in an ENDS product would reduce smokers’ use of cigarettes above and beyond any reduction observed after receiving nicotine from skin patches.

## Methods

### Study design

The study initially employed a 2 × 2 factorial design, with participants randomly assigned to receive nicotine vs. placebo pods to use in their ENDS devices, and nicotine vs. placebo skin patches. Due to unforeseen challenges in participant recruitment, exacerbated by the COVID-19 pandemic, the design was altered mid-study while maintaining the blind and with approval of the funding agency. Participants after this point were enrolled only into the active nicotine patch condition, with randomization to ENDS with vs. without nicotine — the conditions of greatest interest. Additionally, due to COVID-19, study visits were transitioned from in-person to remote format, while maintaining biochemical verification of smoking outcomes. The timeline and key smoking outcomes remained unchanged.

A critically important design component of this study was that it was not a smoking cessation treatment study, and hence smoking abstinence was not the primary outcome. Accordingly, no smoking cessation counseling was provided and smokers who expressed a desire to receive treatment for nicotine dependence were excluded from the study. Thus, the study was not expected to lead to high smoking abstinence rates but was instead designed to differentiate the pharmacologic impact of nicotine vs. no nicotine (in ENDS or patch) on smoking behavior.

### Participant recruitment and screening

Participants were recruited from North Carolina and assessed for eligibility for study participation. Inclusion criteria were as follows: having smoked for the past year; currently smoking at least 10 cigarettes/day; age of 21–65 years; baseline expired air carbon monoxide (CO) reading of at least 10 ppm, body weight <350 lbs., and the ability to read and understand English. Exclusion criteria comprised a number of major medical conditions, illicit drug use or alcohol abuse/dependence, pregnancy, prior adverse reactions to nicotine patch, use of smoking cessation medications within 30 days, use of tobacco products other than cigarettes within 7 days, or anyone seeking treatment for nicotine dependence. Consistent with FDA guidelines (Callahan-Lyon and Sipes 2018), the study was framed as a “switching study,” with the expressed goal of switching from cigarette smoking to ENDS use rather than a “smoking cessation study.” After initial screening on the telephone for inclusion and exclusion criteria, informed consent was obtained and in-person laboratory testing (urine drug screen and pregnancy testing for women) was conducted (either at the clinic or at a local LabCorp laboratory after the study went remote). An interview with a physician assistant provided review of potential participants’ health history. Approval to conduct the study was obtained from the Duke University Medical Center IRB, and participants were compensated for participation.

### Procedure

Individuals who satisfied inclusion/inclusion criteria were enrolled in the study and allocated to one of the following four groups with 2 × 2 factorial randomization: ENDS (with vs. without nicotine); skin patch (with vs. without nicotine). Later in the study, all participants were provided with nicotine skin patches and randomized to ENDS with vs. without nicotine. Participants were provided with study materials (ENDS devices and skin patches) and scheduled for five study visits held at 2-week intervals. The primary study outcome, expired air carbon monoxide, was measured in person in the laboratory, or, after the study went remote, by shipping Bedfont coVita CO breathalyzers to participants with direct observation of CO breath testing through remote televisit (via secure Zoom Videoconference Platform). At each visit, three consecutive CO readings were taken and displayed to the research technician for recording, with the median value used for analyses.

### ENDS

The ENDS product utilized in the study was the JUUL e-cigarette, a breath-actuated, rechargeable closed e-cigarette system. Each pod was pre-filled with 0.7 mL of e-liquid, comprising glycerol, propylene glycol, benzoic acid, flavor, and either 5% nicotine by weight (59 mg/mL) or 0% nicotine (placebo). Nicotine and non-nicotine pods were identical in appearance. JUUL systems were purchased for the study from JUUL Labs (San Francisco, CA), and participants were supplied with two flavors, “Cool Mint” and “Virginia Tobacco.”

### Skin patches

The nicotine skin patch utilized in the study was Nicoderm (GlaxoSmithKline, Philadelphia, PA), delivering 21 mg/24 h or with 0 mg nicotine for controls purchased from Rejuvenation Labs (Salt Lake City, UT). Nicotine and placebo skin patches were identical in appearance.

### Data analysis

Statistical analyses were performed using StatView and SAS software (Cary, NC). The primary outcome was expired air CO at week 8 of the intervention, which was analyzed by analysis of covariance (ANCOVA), with ENDS condition (nicotine vs. placebo) entered as independent variable and baseline CO as a covariate. For the main analysis, only the active patch (21 mg nicotine) condition was used, since enrollment was largely limited to the nicotine patch condition. To confirm the conclusions from the primary analysis, ANOVAs were also conducted on the change in expired CO from baseline to week 8, using data from participants in all four conditions, with ENDS nicotine condition and patch nicotine condition as factors.

The use of 1-tailed or 2-tailed *p* values is specified with reported outcomes. Nicotine, whether administered via ENDS, patches or other means, has only been shown to decrease ad libitum smoking, not to increase it, such that a directional hypothesis was established a priori that nicotine administration from ENDS or patches would reduce (and not increase) smoking. Thus, one-tailed significance testing was used to evaluate whether nicotine vs. placebo ENDS and nicotine vs. placebo patch was associated with reduced expired air CO and cigarettes per day at week 8. When confidence intervals (CI) are given for these comparisons, 90% CIs are provided so that the intervals will correspond to the statistical significance of the one-sided hypothesis test for the effect of nicotine vs. placebo conditions. Given the relatively small number of participants providing CO data in the nicotine patch and placebo patch conditions (*n*=45 and *n*=17, respectively), there was only 54% power to detect a medium effect size (Cohen’s *d*=0.5), and thus negative results observed with the patch must be interpreted with caution.

In addition to the primary outcome of expired air CO, analyses were conducted for the secondary outcome of complete smoking abstinence, defined as a self-report of no smoking, confirmed by expired air CO at week 8 of <5 ppm (Kim et al. 2021). Logistic regression analyses were used for this dichotomous outcome variable and significance assessed with a chi-square likelihood ratio test. Continuous abstinence at weeks 4–8 and point abstinence at week 8 were assessed. Participants who dropped out or could not be reached, or who had missing CO values, were defined as non-abstinent.

Other exploratory analyses examined whether the degree of ENDS or patch use was correlated with the extent of smoking reduction. ENDS and patch use were assessed by self-reports and corroborated by counts of returned (unused) pods and patches.

## Results

### Participants

Table 1 presents the baseline characteristics of the participants in each condition. Overall, the sample comprised 56 males and 38 females (60 white, 28 Black, 6 other, all non-Hispanic), having a mean age of 47.2 years (*SD*=10.3), who smoked 18.5 (*SD*=7.3) cigarettes/day. At baseline, the mean score on the Fagerström Test for Nicotine Dependence (Heatherton et al. 1991) for the study sample was 5.9 (*SD*=2.0) and mean expired CO was 28.9 ppm (*SD*=14.2). There were no significant differences in demographic or smoking history variables between randomized groups.

**Table 1:** Participant characteristics at study entry

Characteristic	Plac pod/Plac patch (*n*=13)	Nic pod/Plac patch (*n*=11)	Plac pod/Nic patch (*n*=33)	Nic pod/Nic patch (*n*=37)
% Male	39.5	45.5	48.5	32.4
% Female	61.5	54.5	51.5	67.6
Race				
% White	46.2	54.6	69.7	67.6
% Black	53.8	45.4	21.2	24.3
% Other	0	0	9.1	8.1
Cigarettes/day (mean ± SD)	17.3 ± 6.3	15.7 ± 8.6	20.3 ± 9.5	19.5 ± 8.7
FIND score (mean ± SD)	6.1 ± 2.2	5.8 ± 1.3	6.1 ± 2.1	5.7 ± 2.1
CO (ppm) (mean ± SD)	28.5 ± 10.7	27.2 ± 13.4	27.7 ± 14.0	30.2 ± 14.3

### Smoking outcomes

**Fig. 1:**
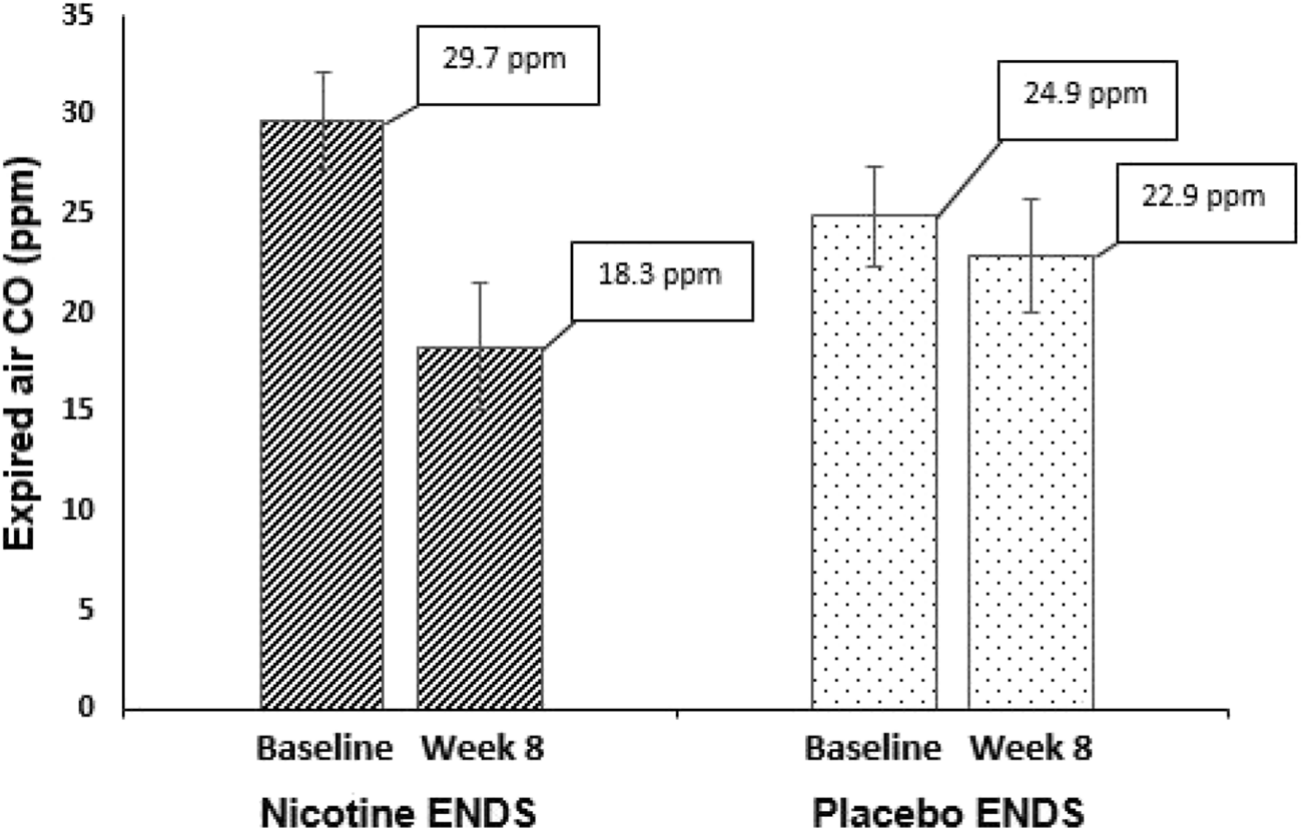
Mean (± s.e.m.) expired air carbon monoxide (CO) readings at baseline and at 8 weeks for nicotine vs. placebo ENDS in daily smokers receiving nicotine patches. The nicotine ENDS condition showed a 42.6% reduction in CO (significant), whereas the placebo ENDS condition showed a 6.4% reduction in CO (nonsignificant)

Among the 45 participants who received active nicotine patches, and from whom CO data were collected at week 8, the ANCOVA analysis showed a significant effect of ENDS with vs. without nicotine on mean expired air CO at week 8 (*B*=−4.2 (*SE*=1.8), *t*=−2.3, *p*=0.01, 1-tailed). Adding covariates of age, race, sex, or baseline FTND score did not change the finding that CO was significantly lower in the nicotine ENDS compared to placebo ENDS condition. In the group receiving nicotine ENDS, the mean CO dropped from 29.7 ppm (*SD*=12.5) at baseline to 18.3 ppm (*SD*= 16.2) at week 8, a mean percent reduction of 42.6% (*SD*=40.5). In contrast, in the placebo ENDS group, expired air CO changed from 24.9 ppm (*SD*=11.0) at baseline to 22.9 ppm (*SD*=12.6), at week 8, with a mean percent reduction of 6.4% (*SD*=49.7), see Fig. 1.

Similar results were obtained from the ANOVA using data from all 62 participants receiving either active or placebo nicotine patches for whom CO data were collected at week 8. There was a significant effect of ENDS nicotine condition (*F*(1,58)=6.41, *p*=0.01, 1-tailed), with no effect of patch condition (*p*>0.9; 1-tailed) and no ENDS x patch interaction (*p*>0.9, 2-tailed). The changes in expired air CO in the four conditions were as follows: nicotine ENDS/nicotine patch (*n*=26): −11.5 ppm (*SD*=12.8); nicotine ENDS/placebo patch (*n*=7): −11.1 ppm (*SD*=16.6); placebo ENDS/nicotine patch (*n*=19): −2.0 ppm (*SD*=10.2); placebo ENDS/placebo patch (*n*=10): −2.2 ppm (*SD*=13.0).

The ENDS nicotine vs. placebo difference in CO change was estimated from the ANOVA model as −9.2 ppm (90% CI (−1.5 ppm, −16.9 ppm)); the patch nicotine vs. placebo difference = −0.1 ppm (90% CI (−7.8 ppm, 7.6 ppm)). The upper bounds of the 90% confidence intervals (pertinent to the directional hypothesis that nicotine would decrease ad libitum smoking) for the effect sizes of CO reduction from ENDS nicotine and patch nicotine were calculated, using a pooled error term from the ANOVA model (pooled standard error=4.7 ppm) and using a noncentral *t* distribution to estimate the CIs. The upper bound of the 90% CI for the patch nicotine effect was *d*=0.47, corresponding to a “medium” effect size, and for the ENDS nicotine, it was *d*=0.84, corresponding to a “large” effect size.

The secondary outcome of self-reported cigarettes/day showed similar trends as did CO. Average cigarettes/day for the last week of treatment (week 8), among 50 participants providing data in the nicotine patch condition, showed significantly lower consumption in the nicotine ENDS condition (ANCOVA, which included baseline consumption and FTND score as covariates: (*B*=−1.5 (*SE*=0.9), *t*=−1.7, *p*=0.04, 1-tailed). Mean daily cigarette consumption in the nicotine ENDS condition decreased from 18.6 cigarettes/day (*SD*=6.9) at baseline to 8.6 cigarettes/day (*SD*=7.7) at week 8. In the placebo ENDS condition, mean daily cigarette consumption decreased from 18.7 cigarettes/day (*SD*=8.9) at baseline to 11.2 cigarettes/day (*SD*=9.3) at week 8. Among 67 participants providing data in all four experimental conditions, the changes in cigarettes smoked per day were as follows: nicotine ENDS/nicotine patch: −10.0 cigarettes/day (*SD*=6.0); nicotine ENDS/placebo patch: −5.6 cigarettes/day (*SD*=3.2); placebo ENDS/nicotine patch: −7.4 cigarettes/day (*SD*=6.9); placebo ENDS/placebo patch: −6.6 cigarettes/day (*SD*=7.2). Although there was a trend for the greatest reduction in cigarette consumption in the condition in which both patch and ENDS delivered nicotine, differences were not statistically significant.

Four-week continuous smoking abstinence, defined as a CO (week 8) < 5 ppm and a self-reported cigarette use of no cigarettes throughout weeks 5–8, was analyzed using logistic regression models and chi-squared test statistic. When examined for all participants (*N*=94), there was a significant effect favoring ENDS nicotine vs. no-nicotine condition (*X*^2^(1, *N* = 94) = 2.82, *p* = 0.046, 1-tailed). There was no significant effect of patch nicotine vs. no-nicotine condition (*X*^2^(1, *N* = 94)=0.2, *p*=0.3, 1-tailed). Abstinence rates were as follows: 10.8% (4/37) in the nicotine ENDS/nicotine patch condition, 9.1% (1/11) in the nicotine ENDS/placebo patch condition, 3% (1/33) in the placebo ENDS/ nicotine patch condition, and 0% (0/13) in the placebo ENDS/placebo patch condition. Point abstinence rates at week 8 showed a similar pattern. There was an effect of nicotine ENDS (*X*^2^(1, *N* = 94) = 2.82, *p*=0.046 (1-tailed) but not for nicotine patch (*X*^2^(1, *N* = 94) = 1.09, *p*=0.15, 1-tailed) and abstinence rates were as follows: 16.2% (6/37) in the nicotine ENDS/nicotine patch condition; 9.1% (1/11) in the nicotine ENDS/placebo patch condition; 6% (2/33) in the placebo ENDS/ nicotine patch condition; and 0% (0/13) in the placebo ENDS/placebo patch condition. Although the effects of patch nicotine content were not significant with the current small sample size, the latter results are consistent with numerically additive effects of ENDS nicotine and patch nicotine.

Time to the first cigarette use of the day was also assessed for potential effects of the independent variables, but there were no significant effects of condition. However, there was a relationship between time to first cigarette and number of ENDS nicotine pods used, as described below.

### Relationship of smoking outcomes to ENDS and patch use

**Fig. 2:**
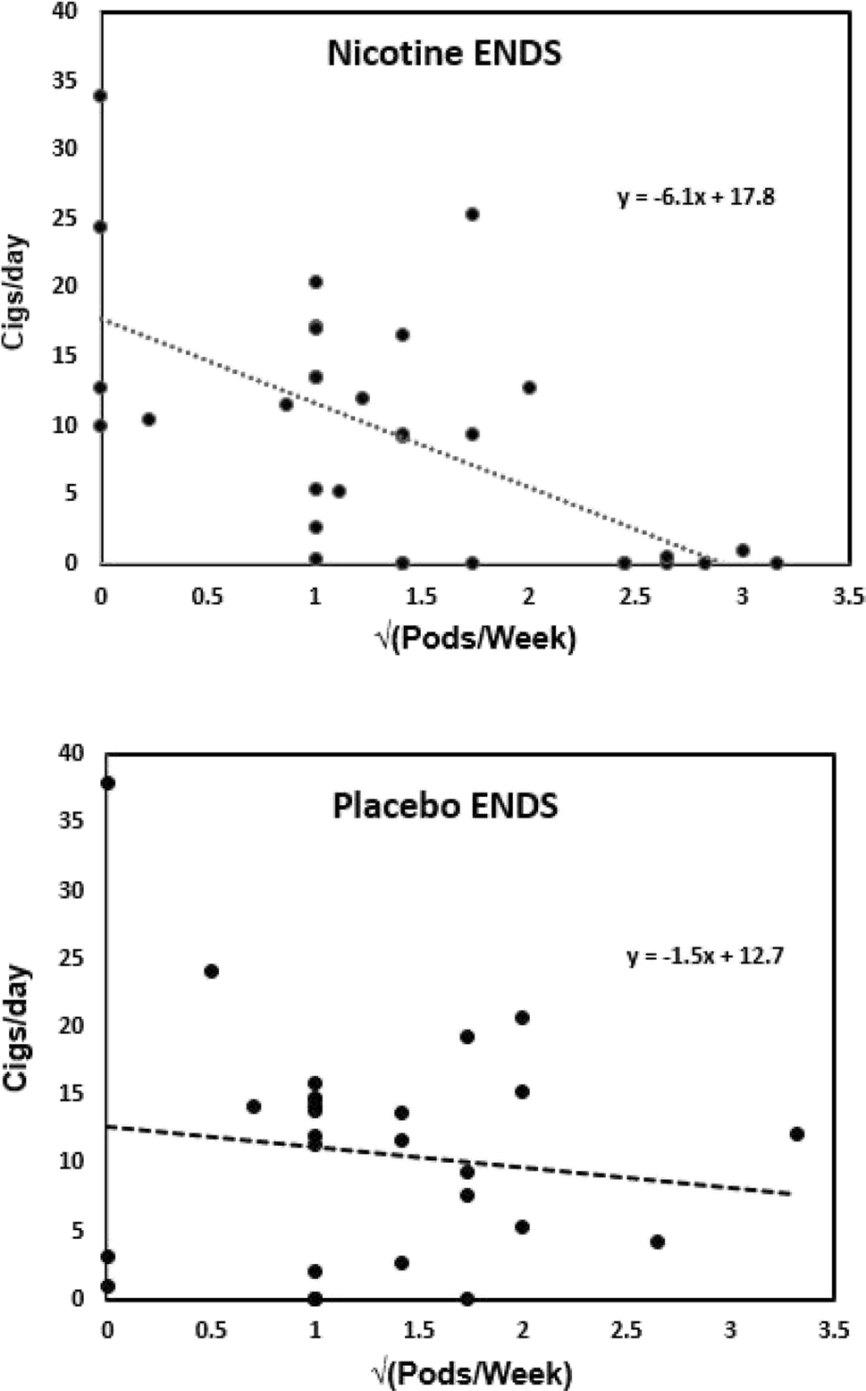
Relationship between ENDS use (pods/week) and cigarettes smoked per day at week 8. A square root transform was used to normalize the skewed distribution of pods/week. Upper panel, nicotine ENDS condition (*r*=−0.61, *p*=0.0002). Lower panel, placebo ENDS condition (*r*=−0.13, *p*=0.52)

As shown in Fig. 2, there was a strong correlation between ENDS use and decrease in self-reported cigarette consumption in week 8 (end of treatment), but only in nicotine ENDS condition: *r*(32)= −0.61, *F*(1,30)=18.01, *p*=0.0002 (2-tailed). The use of placebo ENDS did not show a significant correlation with decrease in cigarette consumption; *r*(28)= −0.13, *F*(1,26)=0.44, *p*=0.52 (2-tailed). A corresponding analysis showed no relationship between nicotine patch use and self-reported cigarette consumption (*p* values>0.5). Similarly, time to the first cigarette of the day was positively associated with ENDS use in the nicotine ENDS condition (*r*= 0.49, *F*(1,22)=6.97, *p*=0.01, 2-tailed) but not the placebo ENDS condition ((*r*= 0.21, *F*(1,22)=1.00, *p*=0.3, 2-tailed). No significant relationships were observed for patch use and time to first cigarette. Smoking abstinence at week 8 was also related to ENDS use in the nicotine ENDS condition only; participants who were abstinent from smoking used more nicotine pods than non-abstinent participants (2.5 pods/week (*SD*=0.6) vs. 1.2 pods/week (*SD*=0.8), *F*(1,30)= 16.58, *p*=0.0003, 2-tailed). Abstinent participants in the placebo ENDS condition used a similar number of pods as non-abstinent participants (1.3 pods/week (*SD*=0.8) vs. 1.4 pods/week (*SD*=0.5); *F*(1,26)=0.04, *p*=0.8, 2-tailed. There was no relationship observed between patch use and smoking abstinence: on average abstinent participants reported using patches on 65% of days vs. 80% for non-abstinent participants (*F*(1,65)=1.27, *p*=0.3, 2-tailed).

### Adverse events

There were several serious adverse events reported by participants during the study, but these were determined by medical review to be unrelated to treatment. These included a fall resulting in knee surgery requiring hospitalization; recurrent COPD exacerbation requiring hospitalization; an infected kidney stone requiring hospitalization; strep throat with pneumonia leading to hospitalization; polysubstance use requiring hospitalization; and hypertensive crisis requiring emergency room treatment in a patient with a known history of poorly controlled hypertension.

## Discussion

The main finding of this study was that the use of nicotine vs. no nicotine in ENDS was associated with a large and statistically significant reduction in expired air CO, an objective biomarker of smoking. The mean reduction in CO was 42.6% for nicotine ENDS vs. 6.4% for placebo ENDS (in the nicotine patch condition). The effect of ENDS nicotine content on self-reported cigarettes/day appeared to be smaller than that for CO, mainly due to a large decline in self-reported smoking in the placebo ENDS condition. Possibly, this decline was offset by compensatory increases in the intensity of smoking each cigarette when the ENDS product did not deliver nicotine, resulting in a disproportionate intake of CO (and presumably other smoke toxins). In any case, CO-verified smoking abstinence rates were significantly higher in the nicotine ENDS condition, and abstinence, self-reported cigarette consumption, as well as time to the first cigarette of the day, correlated with product use in the nicotine ENDS condition, but not in the placebo ENDS condition.

While the smoking abstinence rate in the nicotine ENDS/nicotine patch group of the present study may seem low in the context of smoking cessation treatment trials, it should be borne in mind that this was not a smoking cessation study. There was no smoking cessation counseling and potential participants who were seeking nicotine dependence treatment were excluded from the study. Thus, the study was designed to differentiate the pharmacologic impact of ENDS with nicotine vs. no nicotine on smoking behavior but was not expected to produce high smoking abstinence rates. Studies suggest that rates of quitting smoking among ENDS users who are not motivated to quit smoking are low. Foulds et al. (2022), for example, found that participants receiving a nicotine-containing ENDS (36 mg/mL) showed only 4% point abstinence at 8 weeks, 10.8% point prevalence abstinence at 24 weeks, and 7.7% continuous abstinence of at least 4 weeks at weeks 20 and 24. Interestingly, smoking abstinence rates in that study did increase over time, a finding that has also been reported in a naturalistic study of ENDS use (Goldenson et al. 2021a, b).

The finding that nicotine ENDS use can lead to reductions in ad libitum smoking and promote smoking cessation has been reported in previous studies, but not typically in the context of concurrent nicotine patch use. One exception is a study by Walker et al. (2020), who conducted a pragmatic study of 1124 smokers comparing abstinence rates between groups provided with nicotine patches only, nicotine patches plus nicotine e-cigarettes (18 mg/mL nicotine content), and nicotine patches plus nicotine-free e-cigarettes. There was no placebo patch group as in the present study, but the results were consistent with our finding that nicotine-containing e-cigarettes plus nicotine patches led to the highest abstinence rates. In that study, 3-month self-reported abstinence rates were 33% for nicotine patches plus nicotine e-cigarettes, 23% for nicotine patches plus placebo e-cigarettes, and 18% for nicotine patches only; biochemically confirmed 6-month abstinence rates were 7% for nicotine patches plus nicotine e-cigarettes, 4% for nicotine patches plus placebo e-cigarettes, and 2% for nicotine patches alone.

While the effect of ENDS nicotine content in reducing smoking, assessed by CO, was large, no robust influence of the nicotine content of the patch was apparent. Although the small sample size likely interfered with detecting a modest effect of the nicotine patch, this result is consistent with the results of the Walker et al. (2020) study, as well as other studies that have reported a greater reduction of cigarette use when smokers used ENDS as compared with nicotine replacement (Hajek et al. 2019; Adriaens et al. 2021; McRobbie et al. 2014; Hartmann-Boyce et al. 2022). Likewise, laboratory studies in which nicotine has been administered via skin patches, or via intravenous infusions, have found only small effects on ad libitum smoking behavior (Rose et al. 2003), unless higher doses such as 42 mg or 63 mg are used (Benowitz et al. 1998). There are several reasons why administration of nicotine by inhalation (via ENDS) vs. transdermal administration (via nicotine patches) would lead to greater smoking reduction. First, inhaled nicotine (from cigarettes or ENDS) reaches the bloodstream and brain within seconds, more rapidly than transdermal administration, and within minutes produces higher brain concentrations than transdermal administration, thus providing more immediate reinforcing effects (Rose et al. 2010; Solingapuram Sai et al. 2020). In addition to rapid nicotine absorption, inhaled nicotine produces familiar respiratory tract cues that smokers find rewarding (Rose 2006). These sensory effects of nicotine in ENDS likely contribute to the ability of smokers to substitute ENDS for cigarettes (Di Piazza et al. 2020; Etter 2015; Johnson et al. 2022). Despite providing substitution for behaviors of puffing and inhalation, the use of placebo ENDS does not provide these critical respiratory tract sensations or “throat hit” experienced with aerosolized nicotine, which may account for the observed lack of effect of placebo ENDS in reducing smoking. Thus, although there are several behavioral and sensory components of cigarette smoking, the nicotine-specific pharmacologic effects, potentially including nicotine-associated airway sensory cues, appear to be most important for the ability of ENDS to serve as a substitute for cigarettes.

While the effects of nicotine delivered by ENDS were robust in comparison to those of nicotine patches, there is ample evidence that NRT in the form of nicotine patches can increase smoking abstinence rates in motivated quitters. Although not statistically significant, there were numerically additive effects on point abstinence rates in this study as well as in the Walker et al. (2020) study. Additional studies, perhaps using larger doses of nicotine, may be necessary to clearly demonstrate potentially additive therapeutic effects of transdermal nicotine and nicotine ENDS for cigarette smokers.

Limitations of the study included the modest sample size noted above, which precluded an adequate test of an effect of patch nicotine content on smoking behavior and abstinence. Another limitation was the absence of measures of plasma nicotine or cotinine levels. Without these measurements, we were not able to assess the degree of nicotine replacement achieved through the use of ENDS or through the combined use of ENDS and nicotine patches.

In summary, the results of the study underscore the importance of nicotine in ENDS for suppressing ad libitum cigarette smoking. Specifically, nicotine inhaled from an ENDS device had a robust suppressive effect on smoking behavior, which correlated with the extent of ENDS use.

While using nicotine-containing ENDS did have a marked suppressive effect on smoking, most participants showed “dual use” of cigarettes and ENDS. Additional strategies will therefore need to be developed to facilitate complete switching from cigarettes to ENDS, in order to maximize the potential usefulness of ENDS for tobacco harm reduction.
