# Correction to: Smoking reduction using electronic nicotine delivery systems in combination with nicotine skin patches

**DOI:** 10.1007/s00213-025-06868-x

**Published:** 2025-07-30

**Authors:** Jed E. Rose, Suzanne Frisbee, David Campbell, Alfred Salley, Susan Claerhout, James M. Davis

**Affiliations:** 1 Duke Center for Smoking Cessation, Duke University School of Medicine, 2424 Erwin Road, Suite 201, Durham, NC 27705 USA; 2 Duke Cancer Institute, Durham, NC USA

**Correction to: Psychopharmacology**

10.1007/s00213-023-06401-y

In the published paper, Table 1 listed incorrect number of participants in the four study conditions.

The original article has been corrected.

